# Therapist-Assisted, Internet-Based Treatment for Panic Disorder: Can General Practitioners Achieve Comparable Patient Outcomes to Psychologists?

**DOI:** 10.2196/jmir.1033

**Published:** 2008-05-19

**Authors:** Kerrie Shandley, David William Austin, Britt Klein, Ciaran Pier, Peter Schattner, David Pierce, Victoria Wade

**Affiliations:** ^5^Adelaide Western General Practice NetworkWoodvilleSouth AustraliaAustralia; ^4^School of Rural HealthMelbourne UniversityParkvilleVictoriaAustralia; ^3^Department of General PracticeMonash UniversityNotting HillVictoriaAustralia; ^2^School of PsychologyDeakin UniversityBurwoodVictoriaAustralia; ^1^Faculty of Life and Social SciencesSwinburne University of TechnologyHawthornVictoriaAustralia

**Keywords:** Panic disorder, anxiety, Internet, mental health, general practice, cognitive behavioral therapy, email

## Abstract

**Background:**

Mental illness is an escalating concern worldwide. The management of disorders such as anxiety and depression largely falls to family doctors or general practitioners (GPs). However, GPs are often too time constrained and may lack the necessary training to adequately manage the needs of such patients. Evidence-based Internet interventions represent a potentially valuable resource to reduce the burden of care and the cost of managing mental health disorders within primary care settings and, at the same time, improve patient outcomes.

**Objective:**

The present study sought to extend the efficacy of a therapist-assisted Internet treatment program for panic disorder, Panic Online, by determining whether comparable outcomes could be achieved and maintained when Panic Online was supported by either GPs or psychologists.

**Methods:**

Via a natural groups design, 96 people with a primary diagnosis of panic disorder (with or without agoraphobia) completed the Panic Online program over 12 weeks with the therapeutic assistance of their GP (n = 53), who had received specialist training in cognitive behavioral therapy, or a clinical psychologist (n = 43). Participants completed a clinical diagnostic telephone interview, conducted by a psychologist, and a set of online questionnaires to assess panic-related symptoms at three time periods (pretreatment, posttreatment, and 6 month follow-up).

**Results:**

Both treatments led to clinically significant improvements on measures of panic and panic-related symptomatology from pretreatment to posttreatment. Both groups were shown to significantly improve over time. Improvements for both groups were maintained at follow-up; however, the groups did differ significantly on two quality of life domains: physical (F_1,82_ = 9.13, *P* = .00) and environmental (F_1,82_ = 4.41, *P* = .04). The attrition rate was significantly higher among those being treated by their GP (*χ* ^2^_1_ = 4.40, *P* = .02, N = 96).

**Conclusions:**

This study provides evidence that Internet-based interventions are an effective adjunct to existing mental health care systems. Consequently, this may facilitate and enhance the delivery of evidence-based mental health treatments to increasingly large segments of the population via primary care systems and through suitably trained health professionals.

## Introduction

Projections indicate that by the year 2020 mental health and neurological disorders will account for 15% of the global burden of disease [1]. Such worldwide estimates are comparable in Australia, with mental illness accounting for 13% of total disease burden [2] and 1 in 10 Australian adults reporting that they suffer from a long-term mental or behavioral problem [3].

Despite the high prevalence, up to 40% of people experiencing a mental health problem do not receive any mental health care within a given 12-month period [4]. Typically, when treatment is sought, general practitioners (GPs) are the first, and often only, point of contact [5,6], with a recent Australian national survey finding that psychological problems account for 7.8% of GP visits [7]. Seeking help from a GP confers a number of advantages over other mental health professionals, such as psychologists and psychiatrists, in that GP visits are more accessible, affordable, and less stigmatizing [8].

In an attempt to address gaps in mental health care service provision in Australia, and in recognition of the critical role GPs play in service delivery, the government has expanded the number of Medicare (Australia’s universal health care system) rebate items for mental health consultations, and, in 2001, introduced the Better Outcomes in Mental Health Care (BOiMHC) initiative. The BOiMHC initiative includes educational activities and financial incentives to improve the capacity of GPs to deliver evidence-based psychological interventions such as cognitive behavioral therapy (CBT) [9]. Although the initiative has been welcomed by the health industry and consumers, difficulties in service provision remain. For example, GPs often lack the time and necessary resources and support mechanisms to deliver appropriate psychological interventions to their patients, such as clinical supervision [10]. Consequently, not all GPs and/or regional divisions of general practice choose to participate, thereby creating inequitable patient access. As such, it is important to consider alternative models of delivering effective, evidence-based therapy, particularly for use in primary care. One promising and emerging service delivery modality is the Internet.

### Internet-Based Therapy

Internet-based therapy (eTherapy) typically involves the interaction between a consumer and therapist (eTherapist) via the Internet [11] and incorporates the use of a structured Web-based treatment program for consumers to access in conjunction with eTherapist assistance (usually by email) [12]. Approximately 84% of Australians have access to the Internet [13]; consequently, eTherapy programs offer a unique opportunity to deliver evidence-based mental health treatment, without the need for intensive therapist involvement, to large underserved segments of the population.

Over the past decade, Internet-based treatments have been found effective for a variety of physical health conditions and mental health disorders, such as headache [14], encopresis [15], tinnitus [16], depression [17], and posttraumatic stress symptoms [18]. Based on existing research, the psychological disorder most effectively treated via the Internet is one of the most common anxiety disorders—panic disorder.

### Panic Disorder

Panic disorder affects approximately 1.3% (with agoraphobia, 2.4%) of the Australian population annually [5]. It is characterized by recurrent unexpected panic attacks and is commonly associated with other anxiety disorders [6], depression [19,20], increased risk of suicide [19], and substance and alcohol abuse [20]. Its incidence among people attending general practices has been estimated to be as high as 1 in 12 [21].

CBT is a well established and highly effective treatment for panic disorder (with or without agoraphobia) [22]. The efficacy of CBT for panic disorder appears uncompromised when patients have comorbid depression [23] and when it is transferred from controlled research settings to real-world clinical settings [24]. Although CBT is effective for people with panic disorder, it typically averages 12 hours of face-to-face treatment with a mental health specialist [25]. Furthermore, there are major barriers to accessing expert assistance, including a scarcity of skilled therapists, long waiting lists, high cost, illness symptoms, comorbid conditions, sociodemographic factors, psychological distress, and consumer fears regarding the stigma of a mental health referral [26,27]. These barriers particularly disadvantage people in regional and rural areas where travelling time and distance are an added burden [28].

Internet-based treatments largely address all of these barriers, and, indeed, panic disorder has been effectively treated via the Internet in a number of countries including Sweden [29-31] and the United Kingdom [32]. In Australia, one Internet program for panic disorder, Panic Online (PO), has been developed and extensively evaluated over the past decade.

### Panic Online

Clinical trials have shown that PO, when paired with human support via email (provided by psychologist), is clinically superior to information-only control conditions or other forms of manual and telephone-based therapy [33,34]. PO has also been found to be credible and satisfying to participants [34], and outcomes are unaffected by level of education [35]. Furthermore, a recent exploratory study indicated that PO has the potential to be highly cost-effective [36]. Additionally, PO was recognized by the National Institute of Clinical Studies [37], and notably in a recent meta-analysis, it attained the largest effect size for an Internet-based treatment for a clinical mental health disorder [38].

To our knowledge, PO has not previously been trialled with professional support beyond that of a psychologist, except our own study [39]. This paper reports on the full dataset from a study for which preliminary findings from a limited dataset have been published previously [39]. Given the central role that GPs have in the health system, the aim of the present study was to extend our current understanding of PO’s efficacy by examining participant outcomes when the program is supported by a GP in a traditional face-to-face consultation in comparison to eTherapist assistance.

## Methods

### Participants

A total of 193 people registered for the study, and after 97 were excluded on the basis of inclusion/exclusion criteria, a total of 96 individuals ultimately commenced treatment as part of this study: 43 were recruited into the PO plus psychologist support via email (PO+P) group and 53 into the PO plus face-to-face GP (PO+GP) group.

In total, 132 BOiMHC-trained (CBT-trained) GPs registered to participate in the study, of which 37 actively referred the 53 PO+GP patients and treated participants as per the standardized protocol. Seven psychologists (6 females; 1 male) were employed as eTherapists for the PO+P group and as assessors for both groups.

### Measures

This study utilized three assessment phases (pretreatment, posttreatment, and 6-month follow-up after treatment). Each assessment included a clinical interview conducted over the telephone by a psychologist and the completion of a set of self-administered questionnaires accessed via the Internet. Recent studies have shown that the majority of validated paper-and-pencil questionnaires generally retain their psychometric qualities and produce equivalent results when administered in an online format [40,41].

#### Anxiety Disorders Interview Schedule-IV

 The Anxiety Disorders Interview Schedule-IV (ADIS-IV) is a semistructured clinical interview designed to permit differential diagnosis among anxiety and mood disorders and to screen for other major disorders (eg, substance abuse, psychosis, somatoform disorders). It includes the “number of panic attacks in the last month” (PAMTH). The ADIS-IV has good-to-excellent reliability and validity [42]. In the present study, the ADIS-IV was used to determine eligibility and participant diagnosis at each assessment phase.

#### Anxiety Sensitivity Profile

The Anxiety Sensitivity Profile (ASP) [43] is a 60-item questionnaire measuring the extent to which respondents are fearful that anxiety-related sensations will have harmful consequences. Respondents rate, on a 7-point Likert scale, the extent to which they agree that the sensations described would lead to a bad outcome. The ASP has high test-retest reliability [43].

#### Depression Anxiety Stress Scale

 The Depression Anxiety Stress Scale (DASS) [44] is composed of three 14-item subscales measuring depression, anxiety, and stress. The extent to which a variety of symptoms were experienced within the prior week is rated on a 4-point Likert scale. Alpha coefficients have been reported at .91, .84, and .90 for the depression, anxiety, and stress subscales, respectively [44].

#### Mobility Inventory

The Mobility Inventory (MI) [45] is a measure of agoraphobic avoidance behavior, comprising 27 items. Participants indicate, on a 5-point Likert scale (1 = never avoid to 5 = always avoid), the degree to which they avoid a variety of places or situations when they are alone (MIA) and accompanied (MIB). Acceptable psychometrics have been reported for the MI [45-48].

#### Panic Disorder Severity Scale

The Panic Disorder Severity Scale (PDSS) [49] consists of seven items rated on a 5-point Likert scale (0 = not at all to 4 = most severe). The PDSS is designed to assess the severity of seven dimensions of panic disorder (panic attack frequency, panic attack distress, anticipatory anxiety, agoraphobia fear and avoidance, interoceptive fear and avoidance, occupational impairment/interference, and social impairment/interference) and associated symptoms. The seven items are summed to derive a total score ranging from 0-28, with higher scores reflecting greater symptom severity. The PDSS has excellent interrater reliability and good validity [49].

#### Treatment Credibility Scale-Modified

 The Treatment Credibility Scale-Modified (TCS-M) [50] measures respondents’ attitudes to the credibility of a nominated treatment (in this study, either PO+P or PO+GP). Respondents rate five items on a 10-point scale (0 = not at all to 10 = very much) with respect to how credible they consider their allocated treatment to be after having read a brief rationale and description of the treatment. Higher scores reflect greater levels of perceived credibility.

#### World Health Organization Quality of Life-BREF

The World Health Organization Quality of Life-BREF (WHOQOL-BREF) [51] is a 26-item questionnaire developed from the original WHOQOL 100-item questionnaire. The WHOQOL-BREF covers four domains: physical health (eg, sleep, pain), psychological health (eg, self-esteem, concentration), social relationships (eg, social support, personal relationships), and environment (eg, physical safety, financial resources, recreation).

### Procedure

#### Study Design

The present study employed a natural groups design open to all Australian residents who met the inclusion criteria (detailed below). Participants who were referred to the program by their GP were allocated back to their GP for treatment and were therefore in the PO+GP group. Participants who self-referred to the program (eg, found it via Web surfing, word-of-mouth) were allocated to receive PO supported by an eTherapist and were therefore in the PO+P group.

#### Recruitment

The study was advertised to the general public via participating GPs, Australian mental health websites, and local and national media. Study volunteers could register their interest on the PO website.

GPs were recruited in Victoria, South Australia, and New South Wales via BOiMHC-accredited training programs. Participating GPs were sent a project information package and subsequently were contacted by a research officer (either in person or via telephone) to discuss research protocols, PO program components, the manner in which PO was to be used, and the expected role of the GP and patient in the study. Additionally, regular consultative support was provided by the research officer throughout the duration of the study.

#### Inclusion/Exclusion Criteria

To be included in the study, participants were required to be Australian residents, have computer access, be 18 years or over, be fluent in English, have a primary diagnosis of panic disorder (with or without agoraphobia; as determined via the clinical telephone interview), and to agree not to undertake any other type of therapy for their panic disorder during the study. The request to refrain from other treatments did not cover the follow-up period. At post-assessment, all participants but one (whose data were removed from the analysis) had refrained from other treatments, as measured by self-report.

People were excluded if they reported a seizure disorder, stroke, schizophrenia, hyperthyroidism, organic brain syndrome, heart condition, or chronic hypertension as these are confounding variables with independent associations with panic attacks [52]. People were likewise excluded if they had commenced taking medication in the previous 12 weeks or were not stabilized on their medication dose since this has the potential to confound any treatment effects found for PO.

#### Assessment

Study registrants were contacted by a psychologist who conducted a screening interview to determine whether they met the exclusionary criteria. When exclusionary criteria were met, volunteers were advised of the reason they could not participate and were referred to alternative services as appropriate. When exclusionary criteria were not met, an explanatory statement and consent form were emailed. Upon return of consent, a full clinical diagnostic assessment was conducted via telephone using the ADIS-IV, which took, on average, 90 minutes. Our interrater reliability for this procedure was .93. Following this, participants completed a set of online questionnaires. Upon assessment completion, participants were emailed a username and password with instructions on accessing the PO program. Posttreatment and follow-up assessments (clinical telephone interview and online questionnaires) were conducted at the end of week 12 and 6 months later. Psychologists did not provide therapy for any participant they assessed.

### The Panic Online Program

PO is a 12-week eTherapy program consisting of an introductory module, four learning modules, and a relapse prevention module. The program includes treatment methods commonly used in standard CBT for panic disorder, including instructions for controlled breathing, progressive muscle relaxation, cognitive restructuring, and interoceptive and situational exposure. Downloadable audio of isometric and progressive muscle relaxation and sequential photographic slide shows for two graduated exposure in vivo exercises (going to the supermarket and driving a car) were provided. An adjunct stress management program was also available to all participants (see Richards et al [34]). Information and guidance throughout the program were standardized across participants.

#### Panic Online With Psychologists (PO+P)

Communication between participants and psychologists occurred via email. No limitations were placed on email frequency; however, the assigned eTherapist was instructed to initiate contact if he or she had not received communication from a participant for approximately 1-2 weeks. On average, per participant, eTherapists sent 15.29 emails (SD 9.26; n = 31) and spent 378.62 minutes (SD 264.43; n = 29) emailing participants throughout the 12-week treatment. On average, each eTherapist provided support to 7.17 (range 2-19) participants.

#### Panic Online With General Practitioners (PO+GP)

Following assessment by a psychologist, participants allocated to the PO+GP condition were asked to make an appointment with their GP for their first PO consultation. The GP was then informed by the assessor that the patient could commence treatment. GPs and participants were encouraged to consult regularly (approximately once per week) throughout the treatment duration, while participants were using PO between consultations. On average, participants saw their GP (in a face-to-face consultation) 7.14 times (n = 31) throughout the 12-week treatment.

### Statistical Methods

An independent groups *t* test was conducted to assess treatment credibility. Three repeated measures multivariate analysis of variance (MANOVA), an analysis of covariance (ANCOVA), and an independent groups *t* test were performed to analyze data from this study. MANOVA was conducted to reduce the possibility of type II errors. The first repeated measures MANOVA examined panic symptoms and included the following: clinician-rated panic disorder and agoraphobia severity (as indicated by the ADIS-IV), PAMTH, ASP, and PDSS scores. The second MANOVA examined negative affect and included the three DASS subscales of depression, anxiety, and stress. The final MANOVA examined quality of life and included three of the four WHOQOL-BREF domains (physical, social, and environmental). Lastly, an ANCOVA was conducted to analyze the WHOQOL-BREF psychological domain using the pretreatment assessment score as the covariate. This was analyzed separately as there was a significant difference in the pre-assessment treatment scores between the two groups.

## Results

### Participant Characteristics

In addition to their primary diagnosis of panic disorder, 75 participants were also assessed with clinical levels of agoraphobia (30 in the PO+P group and 45 in the PO+GP group). See Table 1 for a summary of participant characteristics. At pretreatment assessment, 52% of participants were taking medication (19 in the PO+P group and 31 in the PO+GP group). Table 2 provides a breakdown of medication frequencies at pretreatment assessment. Over half of the sample (n = 56) received a secondary clinical diagnosis at pretreatment assessment. Table 3 provides a breakdown of the frequencies of clinically significant comorbid conditions at pretreatment.

**Table 1:** Characteristics of participants at pretreatment assessment, by group

	PO+P			PO+GP			Total		
Characteristic	No.	Mean	SD	No.	Mean	SD	No.	Mean	SD
Age (years)		43.5	12.4		38.7	10.9		40.9	11.8
Education (years)		12.7	2.8		12.9	2.8		12.8	2.8
**Gender**									
Male	10			10			20		
Female	33			43			76		
**Medication**									
Yes	19			31			50		
No	24			22			46		
**Primary diagnosis**									
Panic disorder	13			8			21		
Panic disorder with agoraphobia	30			45			75		
**Clinically comorbid condition at pretreatment assessment**									
Yes	22			34			56		
No	21			19			40		
**Previous mental health treatment (inpatient/outpatient)**									
Yes	20			29			49		
No	23			24			47		

**Table 2:** Medication frequencies at pretreatment assessment, by group

Drug Class^*^	PO+P	PO+GP	Total
SSRI	1	14	15
Benzodiazepine	9	4	13
SNRI	5	2	7
SSRI + Benzodiazepine	2	4	6
Tricyclic antidepressant	1	2	3
Tricyclic antidepressant + SSRI	1	–	1
SSRI + SNRI	–	1	1
Benzodiazepine + SSRI + Antipsychotic	–	1	1
SSRI + Antipsychotic	–	1	1
RIMA + Benzodiazepine	–	1	1
Anticonvulsant + Benzodiazepine + Antipsychotic	–	1	1
**Total**	19	31	50

^*^SSRI, selective serotonin reuptake inhibitor; SNRI, selective noradrenaline reuptake inhibitor; RIMA, reversible inhibitor of monoamine oxidase type A

**Table 3:** Clinical comorbid condition frequencies at pretreatment assessment, by group*

Disorder	PO+P	PO+GP	Total
Generalized anxiety disorder	5	17	22
Depression	9	13	22
Social anxiety disorder	5	15	20
Specific disorder	9	9	18
Dysthymia	4	9	13
Posttraumatic stress disorder	1	6	7
Hypochondriasis	2	4	6
Obsessive compulsive disorder	–	4	4
Alcohol dependence	1	2	3
Substance abuse	–	1	1

^*^Some participants were assessed as having multiple clinical comorbid conditions.

### Attrition

Attrition was defined as participants who withdrew, for reasons either known or unknown, from the research trial. The overall attrition rate for this study was 42.7% (41/96): 37.2% (16/43) and 47.2% (25/53) for the PO+P and PO+GP groups, respectively. This difference was not significant (*χ* ^2^_1_ = .60, *P* = .41, N = 96). Attrition from the treatment and follow-up phase was also examined separately. Overall attrition from pretreatment to posttreatment was 28.1% (27/96), with 16.3% (7/43) dropping out of the PO+P group and 37.7% (20/53) from the PO+GP group. Fisher exact test revealed that significantly more participants in the PO+GP group dropped out of the treatment (*χ* ^2^_1_ = 4.40, *P* = .02, N = 96). A further 14 participants (14.6%) were lost from the study between posttreatment and follow-up assessment. The overall attrition rate from posttreatment to follow-up by condition was 20.9% (9/43) for the PO+P group and 9.4% (5/53) for the PO+GP group; this difference was not significant (*χ* ^2^_1_ = 1.68, *P* = .15, N = 96). Table 4 provides reasons for attrition.

**Table 4:** Reasons for attrition, by group

Reason	PO+P	PO+GP	Total
Unknown	9	9	18
Lost contact	1	4	5
Commencing face-to-face counselling	1	2	3
Computer problems	1	2	3
Personal issues (nonspecific)	2	1	3
GP difficulties		2	2
Cured		1	1
Health problem	1		1
Housing crisis	1		1
Language difficulties		1	1
Moved state		1	1
Personal issues (mental health)		1	1
Pregnancy		1	1
**Total**	16	25	41

### Data Properties and Treatment

This study utilized intention-to-treat analyses. That is, pretreatment assessment scores for participants discontinuing their involvement during treatment were carried forward and used in both the posttreatment and follow-up assessments (11 for the PO+P group; 21 for the PO+GP group). Fisher exact test revealed no difference between the groups (*χ* ^2^_1_ = 1.52, *P* = .19, N = 96). A further 16 PO+P and 13 PO+GP posttreatment assessment scores were carried forward and used in the follow-up assessment. The difference was not significant (*χ* ^2^_1_ = 1.26, *P* = .19, N = 96).

Nonnormally distributed dependent variables were transformed to satisfy normality assumptions. The DASS depression subscale and the MIA required a square root transformation, and PAMTH required a logarithmic transformation.

One-way analysis of variance (ANOVA) tests were conducted on all measures to test for pretreatment differences between groups. A significant pretreatment difference was found in the WHOQOL-BREF psychological domain, with the PO+P group reporting greater quality of life for this domain in comparison to the PO+GP group (Table 5 and Table 6). However, no differences were found between the treatment groups for any other measure (see Table 5). Furthermore, no significant pretreatment assessment differences were detected for age (F_1,94_ = 4.09, *P =* .05), gender (*χ* ^2^_1_ = .08, *P* = .62, N = 96), agoraphobia (*χ* ^2^_1_ = 2.36, *P* = .09, N = 96), medication use (*χ* ^2^_1_ = 1.42, *P* = .22, N = 96), presence of clinically significant comorbid condition (*χ* ^2^_1_ = 1.16, *P* = .22, N = 96), years of education (F_1,81_ = .10, *P* = .75), or previous inpatient or outpatient treatment for a mental health condition (*χ* ^2^_1_ = .35, *P* = .54, N = 96).

Results of evaluation of normality assumptions, homogeneity of variance-covariance matrices, and linearity were satisfactory. Additionally, Bartlett’s test of sphericity was conducted to confirm that the dependent variables in the MANOVA groupings were correlated at the *P* < .05 level. A multivariate outlier was detected in the panic symptoms MANOVA grouping and was subsequently removed due to its impact on the mean.

**Table 5:** *F* ratios and *P* values from pretreatment assessment ANOVA

Variable^*^	*F*	*P*
DASS depression	2.52_1,89_	.12
DASS anxiety	0.72_1,89_	.40
DASS stress	1.60_1,89_	.21
WHOQOL-BREF physical	3.61_1,87_	.06
WHOQOL-BREF psychological	6.09_1,87_	.02
WHOQOL-BREF social	1.94_1,87_	.17
WHOQOL-BREF environmental	2.73_1,87_	.10
MIA	0.41_1,84_	.53
MIB	0.31_1,83_	.58
PAMTH	0.38_1,94_	.54
ASP	0.03_1,88_	.86
PDSS	1.75_1,86_	.19

^*^DASS, Depression Anxiety Stress Scale; WHOQOL-BREF, World Health Organization Quality of Life-BREF; MIA, Mobility Inventory alone; MIB, Mobility Inventory accompanied; PAMTH, panic attacks in the last month; ASP, Anxiety Sensitivity Profile; PDSS, Panic Disorder Severity Scale.

### Treatment Outcomes

#### Treatment Credibility

An independent samples *t* test revealed no significant differences between the groups for perceived treatment credibility prior to treatment (*t*_82_ = 1.96, *P* = .05).

#### Panic Symptoms

For the panic symptoms grouping, repeated measures MANOVA revealed no significant interaction between time (pre, post, follow-up) and group (PO+P, PO+GP) or group main effect. However, a significant main effect for time was found from pretreatment to posttreatment assessment. Examination of the univariate tests for time and associated means revealed a significant decrease on all seven measures. Means and standard deviations are presented in Table 6, multivariate results in Table 7, and univariate results in Table 8.

**Table 6:** Means and standard deviations for treatment outcome measures at pretreatment, posttreatment, and follow-up treatment assessments, by group

	PO+P			PO+GP		
Variable^*^	No.	Mean	SD	No.	Mean	SD
Clinician panic disorder rating						
Pre	43	6.17	1.25	53	6.29	1.29
Post	43	3.43	2.03	53	4.29	2.30
Follow-up	43	3.02	2.42	53	3.84	2.65
Clinician agoraphobia rating						
Pre	43	4.07	2.80	53	5.13	2.35
Post	43	2.16	2.22	53	3.65	2.52
Follow-up	43	2.40	2.34	53	3.40	2.69
PAMTH						
Pre	43	6.33	7.99	53	9.85	14.83
Post	42	2.67	5.48	53	4.27	8.12
Follow-up	42	1.86	2.98	53	4.35	7.93
PDSS						
Pre	38	14.62	4.40	50	16.05	5.45
Post	38	9.71	5.65	52	12.00	6.24
Follow-up	38	9.59	5.96	50	11.73	6.36
ASP						
Pre	39	3.45	1.31	51	3.40	1.42
Post	41	1.88	1.64	51	2.58	1.62
Follow-up	41	1.83	1.61	52	2.50	1.59
MIA						
Pre	41	2.15	.93	45	2.26	.88
Post	39	1.78	.87	40	2.11	.91
Follow-up	37	1.76	.88	44	2.03	.87
MIB						
Pre	41	2.55	1.09	44	2.67	.95
Post	39	2.14	1.08	42	2.36	.95
Follow-up	37	2.16	1.12	45	2.34	.93
DASS depression						
Pre	41	12.24	9.83	50	16.45	12.86
Post	40	7.15	9.76	51	13.52	12.90
Follow-up	41	7.24	9.48	50	12.33	12.54
DASS anxiety						
Pre	41	17.46	10.10	50	19.24	9.80
Post	40	10.28	10.73	51	14.56	10.60
Follow-up	41	10.23	10.33	50	13.64	10.43
DASS stress						
Pre	41	19.26	10.35	50	21.98	10.06
Post	40	12.23	10.84	51	17.24	11.79
Follow-up	41	12.59	10.97	50	16.24	11.51
WHOQOL-BREF physical						
Pre	40	59.05	16.81	49	51.59	19.60
Post	37	69.53	13.85	50	58.54	21.13
Follow-up	38	70.43	14.08	48	57.92	20.94
WHOQOL-BREF psychological						
Pre	40	50.48	18.05	49	41.07	17.76
Post	37	60.47	17.94	50	49.83	18.48
Follow-up	38	60.96	17.45	48	48.83	19.75
WHOQOL-BREF social						
Pre	40	55.00	25.09	49	47.19	27.21
Post	37	61.49	22.85	50	52.17	27.12
Follow-up	38	61.18	22.87	48	50.61	27.64
WHOQOL-BREF environment						
Pre	40	63.38	16.92	49	57.65	15.76
Post	37	67.00	15.01	50	60.58	15.76
Follow-up	38	67.62	15.76	48	60.44	15.27
Treatment credibility						
Pre	39	40.59	7.57	45	37.47	7.02

^*^PAMTH, panic attacks in the last month; PDSS, Panic Disorder Severity Scale; ASP = Anxiety Sensitivity Profile; MIA, Mobility Inventory alone; MIB, Mobility Inventory accompanied; DASS, Depression Anxiety Stress Scale; WHOQOL-BREF, World Health Organization Quality of Life-BREF.

**Table 7:** Effects from the repeated measures MANOVA and ANCOVA analysis between groups*

	Time Effect				Group Effect				Treatment × Time			
Variable	F	*P*	Partial *η*^2^	*β* − 1	F	*P*	Partial *η*^2^	*β* − 1	F	*P*	Partial *η*^2^	*β* − 1
Panic symptoms												
Post	10.28_7,52_	.00	.58	1.00	1.65_7,52_	.14	.18	.62	1.16_7,52_	.35	.14	.45
Follow-up	2.16_7,58_	.05	.21	.77	.90_7,58_	.52	.10	.35	.87_7,58_	.53	.10	.34
Negative affect												
Post	18.04_3,86_	.00	.39	1.0	1.69_3,86_	.48	.06	.42	.80_3,86_	.50	.03	.22
Follow-up	.53_3,86_	.66	.02	.16	.53_3,86_	.10	.07	.52	1.15_3,86_	.33	.04	.30
Quality of life												
Post	15.40_3,82_	.00	.36	1.00	1.95_3,82_	.13	.07	.49	.98_3,82_	.41	.04	.26
Follow-up	.58_3,80_	.63	.02	.17	2.97_3,80_	.04	.10	.68	.01_3,80_	1.00	.00	.05
WHOQOL-BREF psychological												
Post					2.16_1,83_	.15	.03	.31				
Follow-up	.00_1,80_	.95	.00	.05	2.89_1,80_	.09	.04	.39	.23_1,80_	.63	.23	.08

^*^Panic symptoms MANOVA includes clinician-rated panic disorder and agoraphobia severity, PDSS, and PAMTH; negative affect MANOVA includes DASS subscales of depression, anxiety, and stress; quality of life MANOVA includes WHOQOL-BREF physical, social, and environmental domains.

**Table 8:** Effects from univariate tests

	Time Effect				Group Effect			
Variable	F	*P*	Partial *η*^2^	*β* − 1	F	*P*	Partial *η*^2^	*β* − 1
**Pretreatment to Posttreatment**								
Panic disorder	69.49_1,58_	.00	.55	1.00				
PAMTH	29.91_1,58_	.00	.34	1.00				
ASP	35.37_1,58_	.00	.46	1.00				
PDSS	50.14_1,58_	.00	.46	1.00				
Agoraphobia	37.23_1,58_	.00	.39	1.00				
MIA	15.16_1,58_	.00	.21	.97				
MIB	21.79_1,58_	.00	.27	1.00				
DASS depression	41.18_1,88_	.00	.32	1.00				
DASS anxiety	47.98_1,88_	.00	.35	1.00				
DASS stress	44.66_1,88_	.00	.34	1.00				
WHOQOL-BREF physical	45.91_1,84_	.00	.35	1.00				
WHOQOL-BREF social	9.98_1,84_	.00	.11	.88				
WHOQOL-BREF environmental	12.07_1,84_	.00	.13	.93				
**Posttreatment to Follow-Up**								
WHOQOL-BREF physical					9.13_1,82_	.00	1.00	.85
WHOQOL-BREF environmental					4.41_1,82_	.04	.05	.55

^*^PAMTH, panic attacks in the last month; ASP, Anxiety Sensitivity Profile; PDSS, Panic Disorder Severity Scale; MIA, Mobility Inventory alone; MIB, Mobility Inventory accompanied; DASS, Depression Anxiety Stress Scale; WHOQOL-BREF, World Health Organization Quality of Life-BREF.

#### Negative Affect

For the negative affect grouping, repeated measures MANOVA revealed no significant interaction between time and group or group main effect. However, a significant main effect for time was found from pretreatment to post treatment assessment (see Table 7). Examination of the univariate tests for time (see Table 8) and associated means (see Table 6) revealed a significant decrease on all three DASS subscales.

#### Quality of Life

For the quality of life grouping, repeated measures MANOVA revealed no significant interaction between time and group. However, a significant main effect for time from pretreatment to posttreatment assessment and a significant main effect for group from posttreatment to follow-up assessment were found (see Table 7). Examination of the univariate tests for time (see Table 8) and associated means (see Table 6) revealed a significant positive change on all three domains from pretreatment to posttreatment. Examination of the univariate between-subject effects from posttreatment to follow-up revealed a significant difference between the groups for the WHOQOL-BREF physical and environmental domains. The mean scores for both domains (see Table 6) showed that the PO+P group experienced a slight improvement, whereas the PO+GP group showed a slight decrease from posttreatment to follow-up.

#### WHOQOL-BREF (Psychological)

An ANCOVA was conducted on the psychological domain of the WHOQOL-BREF from pretreatment to posttreatment and posttreatment to follow-up. No significant differences were detected (see Table 7).

#### Panic-Free Status and High-End State Functioning

Panic-free status and high-end state functioning were examined at posttreatment and follow-up assessment. Panic-free status was defined as zero panic attacks reported during the month immediately prior to the assessment. At posttreatment assessment, panic-free status was achieved by 52.4% (22/42) of the PO+P group and 50.9% (27/53) of the PO+GP group; this difference was not significant (*χ* ^2^_1_ = .00, *P* = 1.00, N = 95). At follow-up, 52.4% (22/42) of the PO+P group and 47.2% (25/53) of the PO+GP group were panic free, but this difference was also not significant (*χ* ^2^_1_ = .09, *P* = .68, N = 95).

High-end state functioning was defined as being panic free and having a clinician-rated panic disorder score ≤ 2. At posttreatment assessment, 28.6% (12/42) of the PO+P group and 26.4% (14/53) of the PO+GP group achieved high-end state functioning, but this difference was not statistically significant (*χ* ^2^_1_ = .00, *P* = .82, N = 95). At follow-up, 47.6% (20/42) of the PO+P group and 32.1% (17/53) of the PO+GP group achieved high-end state functioning, but again, the difference was not significant (*χ* ^2^_1_ = 1.77, *P* = .14, N = 95). However, for the PO+P group, the increase in high-end state functioning from posttreatment to follow-up was significant (*t*_41_ = −2.44, *P* = .02).

## Discussion

The purpose of the current study was to investigate whether the established efficacy of PO was affected by changing the form of therapist assistance from email support provided by psychologists (eTherapists) to face-to-face support provided by GPs, and, further, whether treatment improvements were maintained. The results of this study support findings from several previous studies examining Internet programs in primary care [53,54] and demonstrate that evidence-based eTherapy programs could be a valuable tool for GPs managing patients with mental health conditions.

The recommended treatment for panic disorder includes CBT, medication (antidepressants and/or benzodiazepines), or a combination of both [55]. However, there are difficulties associated with each form of treatment. Barriers such as accessibility, waiting lists, cost, and stigma inhibit access to CBT experts [26,27], and use of pharmacotherapy is often complicated by side effects, compliance, and other health considerations [56]. Furthermore, medication use in comparison to CBT treatment does not appear to result in sustained recovery beyond discontinuation [56,57]. Consequently, investigating other methods of delivering cost-effective and clinically effective treatment is important to address the growth of mental health disorders both in general practice and the wider community.

In this study, PO (whether supported by eTherapists or face-to-face GPs) led to significant improvements in panic attack frequency, depression, anxiety, stress, anxiety sensitivity, agoraphobia avoidance, and quality of life. Improvements were maintained at follow-up, with the only significant differences occurring on the WHOQOL-BREF physical and environmental domains. It is beyond the capacity of this study to ascertain definitively why the groups differed on these particular measures. It is possible to speculate, however, that the different dissemination processes (email vs face-to-face) created disparate learning experiences between the groups, resulting in the PO treatment information being used and retained in different manners. Further, while the groups did not significantly differ on any pretreatment assessment sociodemographic measure, the PO+GP group did have a higher degree of comorbidity and proportion of participants on medication. Consequently, it is possible that this study inherently measured two different cohorts.

Surprisingly, attrition from treatment was significantly higher for the PO+GP group. A number of possible reasons can be hypothesized. First, there was variation in the level of support throughout the duration of the trial. While participants in the PO+GP group were encouraged to regularly access their GP throughout treatment, this was not a requirement, and GP visitations could not be reasonably regulated within this study. By contrast, participants in the PO+P group were able to email their therapist as often as they wished, and their therapist was required to respond within 24 hours. Second, greater effort and planning are required to attend a medical practice in comparison to writing an email. Consequently, participants in the PO+P group may have experienced a greater level of continuous support and encouragement to adhere to the treatment. It is also worth noting that while treatment credibility was not significantly different between the groups, it did near significance, with the PO+P treatment appearing to be viewed more favorably than the PO+GP treatment. Finally, it is not known whether the content of GP visits focused specifically on panic disorder or incorporated consultation on other unrelated ailments. However, in comparison to other Internet-based studies [14,16], attrition overall in this study was relatively low.

It is noteworthy that the proportion of participants achieving high-end state functioning in both groups continued to increase from posttreatment to follow-up and that for the PO+P group, the increase was significant. These results not only support the durability of PO to maintain treatment outcomes but also indicate that it has the capacity to continue to have benefits beyond treatment completion.

### Limitations

There are several methodological issues and limitations to note. The primary limitation of this study was that it used a nonrandomized, natural groups design. Consequently, we can not speak to the direct comparability of these two treatments, and it is possible that the groups differed in ways not considered within this study. It should also be mentioned that all participating GPs were trained in delivering CBT. It is unknown whether non-CBT-trained GPs would achieve similar outcomes. This issue would benefit from further investigation as the accessibility to the program would be increased substantially if the evidence base indicated that all GPs were able to effectively support patients using the program. As discussed earlier, the treatments differed in terms of the supportive communication modality employed. This factor may have affected attrition and was not investigated. A final issue relates to PO access. Unfortunately, participant usage statistics (eg, number of times accessed PO, duration of time spent on PO) were not available. Consequently, it is possible that one group may have spent a proportionally greater period of time accessing and/or reading the PO material and therefore achieved and sustained greater benefits.

### Implications

A number of implications for policy and practice can be derived from this study. While it is anticipated that there might be reluctance to adopt eTherapy into general practice [58], this study has demonstrated the capacity of evidence-based programs, such as PO, to aid GPs in the management of mental health disorders, such as panic disorder, and achieve sustained outcomes, making them an invaluable tool. However, at present, there is no specific Medicare and/or private health insurance rebates on such services. Furthermore, there is need for appropriate educational and financial support within primary care to integrate these programs within existing public health systems.

### Conclusions

This study demonstrates that when panic disorder sufferers are provided with accessible online treatment protocols, CBT-skilled GPs can achieve sustained patient outcomes comparable to best-practice treatments delivered by psychologists. Further research will be required to evaluate Internet-based programs for other mental health conditions and with non-CBT-trained GPs. Nevertheless, this study provides strong evidence that the use of Internet-based programs is an effective adjunct to existing mental health care services and may enable the delivery of evidence-based treatments to increasingly large numbers of patients via primary care with the support of suitably trained health professionals.

## Abbreviations

ASP: Anxiety Sensitivity Profile
BOiMHC: Better Outcomes in Mental Health Care
CBT: cognitive behavioral therapy
DASS: Depression Anxiety Stress Scale
GP: general practitioner
MIA: Mobility Inventory alone
MIB: Mobility Inventory accompanied
PAMTH: panic attacks in the last month
PDSS: Panic Disorder Severity Scale
PO: Panic Online
WHOQOL-BREF: World Health Organization Quality of Life-BREF
